# Saliva Cortisol and Exposure to Aircraft Noise in Six European Countries

**DOI:** 10.1289/ehp.0900933

**Published:** 2009-07-20

**Authors:** Jenny Selander, Gösta Bluhm, Töres Theorell, Göran Pershagen, Wolfgang Babisch, Ingeburg Seiffert, Danny Houthuijs, Oscar Breugelmans, Federica Vigna-Taglianti, Maria Chiara Antoniotti, Emmanuel Velonakis, Elli Davou, Marie-Louise Dudley, Lars Järup

**Affiliations:** 1 Institute of Environmental Medicine, Karolinska Institutet, Stockholm, Sweden; 2 Stress Research Institute, Faculty of Social Sciences, Stockholm University, Sweden; 3 Department of Environment and Health at the Federal Environmental Agency, Berlin, Germany; 4 National Institute of Public Health and Environmental Protection, Bilthoven, the Netherlands; 5 Environmental Epidemiologic Unit, Regional Agency for Environmental Protection, Piedmont Region, Grugliasco, Italy; 6 Department of Prevention, Azienda Sanitoria Locale 13 Novara, Novara, Italy; 7 Laboratory of Prevention, Nurses School, National and Kapodistrian University of Athens, Athens, Greece; 8 Department of Hygiene and Epidemiology, National and Kapodistrian University of Athens, Athens, Greece; 9 Medical Research Council and the Health Protection Agency, Centre for Environment and Health, Department of Epidemiology and Public Health, Imperial College London, London, United Kingdom

**Keywords:** cardiovascular disease, gender differences

## Abstract

**Background:**

Several studies show an association between exposure to aircraft or road traffic noise and cardiovascular effects, which may be mediated by a noise-induced release of stress hormones.

**Objective:**

Our objective was to assess saliva cortisol concentration in relation to exposure to aircraft noise.

**Method:**

A multicenter cross-sectional study, HYENA (Hypertension and Exposure to Noise near Airports), comprising 4,861 persons was carried out in six European countries. In a subgroup of 439 study participants, selected to enhance the contrast in exposure to aircraft noise, saliva cortisol was assessed three times (morning, lunch, and evening) during 1 day.

**Results:**

We observed an elevation of 6.07 nmol/L [95% confidence interval (CI), 2.32–9.81 nmol/L] in morning saliva cortisol level in women exposed to aircraft noise at an average 24-hr sound level (*L*_Aeq,24h_) > 60 dB, compared with women exposed to *L*_Aeq,24h_ ≤ 50 dB, corresponding to an increase of 34%. Employment status appeared to modify the response. We found no association between noise exposure and saliva cortisol levels in men.

**Conclusions:**

Our results suggest that exposure to aircraft noise increases morning saliva cortisol levels in women, which could be of relevance for noise-related cardiovascular effects.

Transportation noise is a significant and increasing problem in urban areas worldwide (World Health Organization 2000). There is mounting evidence of an association between road traffic as well as aircraft noise and cardiovascular outcomes (Babisch 2006; Babisch et al. 2005; Belojevic et al. 2008a; Bluhm et al. 2007; de Kluizenaar et al. 2007; Eriksson et al. 2007; Rosenlund et al. 2001; van Kempen et al. 2002; Willich et al. 2006). One proposed biological mechanism implies that noise causes a release of stress hormones, which in turn adversely affect cardiovascular risk factors (Babisch et al. 2001; Ising and Kruppa 2004; Spreng 2000). An intermediary mechanism may involve the metabolic syndrome in which a disturbed hypothalamus–pituitary–adrenal axis regulation has been assumed to play an important role (Chandola et al. 2006). The glucocorticoid hormone cortisol is the main secretory product of the neuroendocrine cascade and a valid indicator of stress (Evans et al. 2001; Miki et al. 1998; Schulz et al. 1998). The cortisol profile normally shows a diurnal variation, high in the morning and low at night (Hofman 2001). After long-time stressful exposure, the ability to down-regulate cortisol may be inhibited (Spreng 2000).

Stress hormone studies on community noise exposure have generally been performed using urine and blood measurements (Babisch 2003; Babisch et al. 2001; Dallman 1993; Evans et al. 2001; Maschke 2003; Miki et al. 1998; Pruessner et al. 1999; Schulz et al. 1998). Saliva cortisol measurements are easy to perform, reliably reflect free cortisol levels in blood (Hofman 2001), and have recently been used in a few studies on exposure to road traffic and aircraft noise (Poll et al. 2001; Stansfeld et al. 2001; Waye et al. 2003). In two review articles, the relationship between road traffic noise as well as aircraft noise and cortisol was investigated (Babisch 2003; Ising and Kruppa 2004). Six of the 14 studies reviewed showed an increase in cortisol level related to exposure, but these were mainly based on urine measurements and had small sample sizes. Only two of the studies used saliva cortisol measures, and the results were inconclusive (Poll et al. 2001; Stansfeld et al. 2001). Thus, the association between community noise exposure and cortisol levels is still unclear, particularly regarding exposure–response relationships and gender differences.

The HYENA (Hypertension and Exposure to Noise near Airports) multicenter study, which included six European countries, revealed an association between nighttime aircraft noise as well as average daily road traffic noise exposure and risk of hypertension (Jarup et al. 2008). An acute blood pressure increase was also related to aircraft or road traffic noise in a subsample (Haralabidis et al. 2008). Our objective was to study saliva cortisol as a possible marker of noise-induced stress in a subgroup from the HYENA project.

## Materials and Methods

### Study subjects

The HYENA study was based on seven airports in six countries: United Kingdom (Heathrow), Germany (Tegel), the Netherlands (Schiphol), Sweden (Arlanda and Bromma), Greece (Athens), and Italy (Malpensa). For the main study, men and women 45–70 years of age living in selected areas surrounding these airports were invited. A total of 4,861 subjects (2,404 men and 2,457 women) participated. The participation rate differed among countries, from about 30% in Germany, Italy, and the United Kingdom to 46% in the Netherlands, 56% in Greece, and 78% in Sweden. A nonresponder analysis showed no significant differences in occurrence of cardiovascular risk factors between participants and nonresponders (Jarup et al. 2008). Men and women who had lived at their address for < 5 years or lived at their address < 6 months each year were excluded from the main study, as were subjects who were too ill to participate or could not comprehend the questionnaire.

The sample for the saliva study was drawn from the HYENA main study. All participants were eligible for saliva sampling except shift workers, who were excluded because cortisol levels are related to the circadian rhythm and vary with the work schedule. To increase contrast in exposure, participants with the highest and lowest levels of exposure to aircraft noise in each country were selected for saliva sampling. A total of 84 subjects from each of the six participating countries were to provide saliva samples with the aim to recruit a total number of 500 participants. The participation rate was high in Sweden, the United Kingdom, and the Netherlands (99%, 98%, and 85%), lower in Italy (49%) and Germany (26%), and was not recorded in Greece. A final sample of 439 participants (209 men and 230 women) participated (Table 1). The study was approved by ethical committees in each of the participating countries, and the participants gave their informed consent before the study.

### Assessment of noise exposure and risk factors

The study subjects were interviewed at home by a nurse using a standardized questionnaire with focus on known risk factors for cardiovascular disease such as health status and socioeconomic and lifestyle factors (diet, physical activity, smoking habits). The participants also underwent blood pressure measurements, and height and weight were assessed to calculate body mass index (BMI). This procedure is described in detail elsewhere (Jarup et al. 2005)

Aircraft noise exposure was assessed for each participant’s home address using noise maps from each of the participating countries (Jarup et al. 2005). Briefly, the home addresses were transformed into coordinates and marked on a geographic information system map with contours in 1-dB intervals for aircraft noise. The dB levels were calculated using three different periods of the day and aircraft noise data from the year 2002. This resulted in the following five variables: average sound level for 24 hr (*L*_Aeq,24h_); average sound level for 24 hr, +5 dB in evening, +10 dB at night (*L*_den_); average during the day (*L*_day_); average during the evening (*L*_evening_); and average during the night (*L*_night_). The noise maps were calculated with the integrated noise model (Gulding et al. 1999) in all participating countries, except for the United Kingdom, where the Ancon model was applied (Ollerhead et al. 1999).

For road traffic noise, local models were used. The Good Practice Guide for Strategic Noise Mapping (Working Group Assessment of Exposure to Noise 2006) was used to merge the data between countries. In most countries, only 24-hr data on the intensity of road traffic were available. The variables *L*_Aeq,24h_ and *L*_night_ were both derived from these data, so no distinction could be made between the relative effects on cortisol level of road traffic noise exposure during the night or during the day (Jarup et al. 2005).

### Cortisol measurements

The participants selected for saliva sampling received a kit with three test tubes, instructions, cover letter, and return envelope. The subjects were instructed to collect the first sample 30 min after awakening (which usually corresponds to the peak excretion of cortisol), the second sample immediately before lunch, and the third sample just before going to bed in the evening. Tooth brushing, smoking, and food and drink intake were to be avoided 30 min before sampling. Detailed instructions were given to the participants to ensure that the samples were taken in a similar fashion for all participants in all countries and that the samples would be marked properly.

Each of the test tubes included a small cotton swab. The participants were instructed to put the swab in their mouth until the swab was completely soaked by saliva and then place it in the test tube, write the date and time on the label of the tube, and put it in the refrigerator. After all samples were taken, the test tubes were either returned by post or collected by a fieldworker.

The samples were first centrifuged and frozen in a laboratory in each of the participating countries. When all samples had been received in each country, respectively, the saliva tubes were sent to a laboratory at Karolinska Institutet (Stockholm, Sweden) for analysis.

Cortisol levels in saliva were determined by the Spectria cortisol coated tube radioimmuno-assay kit (Orion Diagnostica, Espoo, Finland). All samples from each subject or group of subjects were analyzed simultaneously in duplicate. The within- and between-assay coefficient of variation never exceeded 5.0% and 10.0%, respectively. Cortisol was analyzed and compared for 30 samples at the Department of Physiological Psychology, University of Düsseldorf, Germany (Kirschbaum and Hellhammer 1999). There was a very high correlation (0.98) but a slight difference in level, with systematically lower levels in the Stockholm laboratory. The difference was 12.5%, with 95% confidence interval (CI) of 1.5–22.3%.

### Statistical analyses

We analyzed associations between noise exposure and saliva cortisol levels using linear regression models, including interaction terms between covariates. Results are expressed mainly as regression coefficients and 95% CIs. A covariate was selected as a confounder if the inclusion of this variable in the preliminary model changed the regression coefficient for noise exposure by > 10%. A full model was then created, and the covariates that affected the full model < 3% were discarded in the final model.

The covariates included in the final fully adjusted regression model were aircraft noise (continuous), road traffic noise (continuous), country (six categories), age (continuous), sex (dichotomous), BMI (continuous), alcohol use (continuous), diet (nine categories based on vegetable and fruit intake), employment status (three categories: employed, retired, other), occupational status (five categories: lower manual to higher managerial), noise-reducing actions during the night (dichotomous), and medication use (dichotomous). Four nighttime annoyance variables (railway, construction, industry, and indoor) were categorized in accordance with the International Commission on Biological Effects of Noise 11-category scale (Fields et al. 2001). In the aircraft annoyance analyses, the variable was classified in three categories (low, 0–3; moderate, 4–7; high, 8–10). All information about potential confounders except for road traffic noise exposure was obtained from the questionnaire. Heterogeneity tests revealed no statistically significant differences between countries, and fixed-effect models were used in the combined analyses.

The statistical analyses were performed with STATA version 8.2 (StataCorp., College Station, TX, USA).

## Results

**Figure 1:**
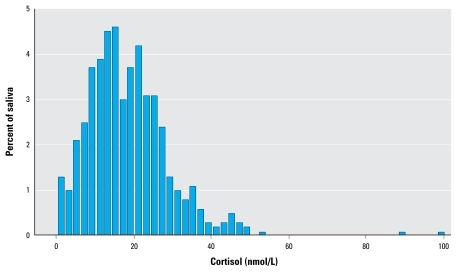
Distribution of cortisol for morning saliva samples from 439 participants exposed to aircraft noise in six European countries.

The cortisol levels in the morning sample showed a roughly normal distribution (Figure 1). We observed two “outliers” in the morning samples, one in the United Kingdom (99.8) and one in Sweden (89.0). However, these values were still within the normal range and were therefore included in the subsequent analyses.

**Figure 2:**
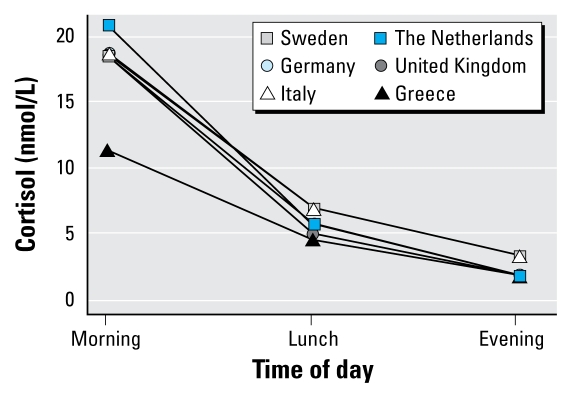
Median cortisol level for each country for morning, lunch, and evening saliva samples.

The median levels for morning, lunch, and evening samples were comparable across countries (Figure 2), except for Greece, which had a lower median level for the morning sample.

Table 2 shows linear regression coefficients and 95% CIs for cortisol level in saliva from the morning sample in relation to aircraft noise levels (*L*_Aeq,24h_). For women we found a significant association between aircraft noise exposure and morning saliva cortisol levels in the analysis, based on a continuous exposure variable. This corresponds to a 5% increase in mean cortisol level for each 5-dB rise in exposure. Women experiencing an aircraft noise level > 60 dB had a significantly higher morning saliva cortisol concentration than did women with aircraft noise exposure < 50 dB; the difference in means was 6.07 (95% CI, 2.32–9.81) nmol/L, corresponding to a 34% increase. For men, we found no association between aircraft noise and cortisol levels. Separate analyses without the previously mentioned outliers (Figure 1) did not change the results.

In country-specific analyses, we found a statistically significant increase in morning cortisol levels in women exposed to air traffic noise only for the United Kingdom (Heathrow), but we found no heterogeneity in results among the countries (Table 3). No clear effects were indicated among men. The number of investigated subjects for either sex in each country was small, leading to substantial statistical uncertainty of the estimates.

Women exposed to aircraft noise > 60 dB had an increase in morning saliva cortisol level regardless of whether they considered themselves annoyed. We found a 25% increase (4.86 nmol/L; 95% CI, –0.46 to 10.18 nmol/L) in mean saliva cortisol for those who reported low annoyance, 52% increase (9.96 nmol/L; 95% CI, 3.30–16.62 nmol/L) for moderately annoyed participants, and a 30% increase (5.73; 95% CI, 0.05–11.40 nmol/L) for highly annoyed study subjects compared with participants with an exposure < 50 dB and who reported low annoyance. We found no rise in saliva cortisol level among participants who reported moderate or high annoyance in the lowest exposure group, < 50 dB.

Employed women had higher morning saliva cortisol levels than did retired women, particularly among those with high exposure to aircraft noise (Table 4). Women exposed to aircraft noise > 60 dB and who were currently employed had an 83% higher mean morning saliva cortisol level [16.23 (95% CI, 9.29–23.2) nmol/L] compared with retired women exposed to aircraft noise level at ≤ 50 dB. Retired women exposed to aircraft noise > 60 dB had an increase of 27% [5.38 (95% CI, −0.57 to 11.33) nmol/L. Additional analysis restricted to participants ≥ 55 years of age showed a similar increase, indicating that the effect is not explained by age differences.

The relation between aircraft noise and cortisol did not change noticeably using the five different aircraft noise variables. In principle, night flights may be of great importance for the morning saliva levels. However, morning flights might also affect the morning saliva levels but are not included in the *L*_night_ estimates. Limited night flights occurred for Tegel Airport in Berlin (no flights between 2200 hr and 0500 hr) and no night flights occurred for Bromma Airport in Stockholm. Thus, we used imputed values for *L*_night_ estimates, which may contribute to a greater uncertainty of the estimates. We also performed analyses using all saliva samples, including differences between day or evening and morning levels, but we found no consistently different associations compared with those presented here.

We found no apparent associations between road traffic exposure and saliva cortisol levels.

## Discussion

The main finding in this study was a significant exposure–response increase in cortisol levels in the morning sample for women exposed to aircraft noise. The HYENA main study found a significant exposure–response relationship between nighttime aircraft noise exposure and risk of hypertension (Jarup et al. 2008). This agrees with our findings in women, although we found no comparable association in men. The differences in noise influence on saliva cortisol between men and women are difficult to explain. However, it is of interest that recent studies have shown a relation between occupational stress and elevated morning saliva cortisol concentration only in women (Alderling et al. 2006; Lundberg 2005; Maina et al. 2009; Rystedt et al. 2008). Previous studies regarding traffic noise exposure in relation to hypertension or myocardial infarction have been inconclusive regarding sex differences (Babisch et al. 2005; Belojevic et al. 2008b; Jarup et al. 2008; Selander et al. 2009; Willich et al. 2006).

We found no consistent effect of road traffic noise on morning saliva cortisol levels, although HYENA study found a significant association between average daily road traffic exposure and hypertension, primarily among men (Jarup et al. 2008). One explanation could be that the noise exposure contrast in our study was less for road traffic noise than for aircraft noise as a result of our selection focusing on subjects with high and low exposure to aircraft noise. Because we investigated only a subgroup from the main study, this leads to less powerful analyses, which may contribute to the apparent lack of association.

The focus in this study was not on pathologic or abnormal saliva cortisol levels but rather on differences in distribution of values in different exposure groups. In individual cases, at least five samples during the day and at least three sampling days would be needed for a reliable individual assessment of saliva cortisol. On a group level, however, three samples per person during one sampling day may be sufficient (Bigert et al. 2005). The first rise in the morning 30 min from awakening may be particularly relevant because it may reflect long-lasting stress levels (Kirschbaum and Hellhammer 1999; Kudielka and Kirschbaum 2003).

The country-specific analyses suggested a stronger association between morning cortisol levels and exposure to aircraft noise in the United Kingdom (Heathrow), although no statistically significant heterogeneity was indicated between the countries. London’s Heathrow is a major airport, with night flights and residents living close to the runways, whereas in the other countries restrictions in night traffic, smaller airports, and/or rural residential areas next to the airport contribute to lower exposure levels. Among the participants in Greece, the mean level of cortisol in the morning sample was lower than in the other countries, possibly because the Athens International Airport is new and the participants had been exposed for a much shorter time than the participants in the other countries. It is biologically plausible that a longer exposure time is needed to develop chronic stress reaction with increase in salivary cortisol levels. The response rates for the saliva sampling differed across countries. In Germany, Greece, and Italy, the response rate was low or not recorded. This can contribute to a lower validity, but it is uncertain how this affected the results.

In our study, annoyance to aircraft noise did not seem to have a relation to morning saliva cortisol level. We noted a rise in saliva cortisol with increasing noise levels regardless of the annoyance level, and the highest exposure group (> 60 dB) showed an increase in saliva cortisol even among participants with low annoyance. In contrast, participants in the lowest exposure group showed no change in cortisol level even if they were highly annoyed. These findings suggest that the effect is not dependent on the subjective annoyance experience but rather is connected directly to noise exposure.

It is also of interest that women who were employed had a higher cortisol level than did retired women. We found a particularly strong increase in employed women exposed to high levels of aircraft noise. This effect could be a result of disrupted sleep during night and the lack of recovery during the day due to employment. It can also be a result of stressful activities related to employment (Alderling et al. 2006; Lundberg 2005; Maina et al. 2009; Rystedt et al. 2008) combined with aircraft noise exposure at home. Further variables that may influence the results are marital status and number of children. Unfortunately, we lacked information regarding these factors, but adjusting for number of occupants in the participant’s household did not change the estimates markedly.

In conclusion, we found a significant increase in cortisol levels in the morning for women exposed to aircraft noise, but no comparable association in men. Our results provide some support for a physiologic stress reaction induced by noise, which may contribute to hypertension and other adverse cardiovascular effects.

## Figures and Tables

**Table 1:** Number of participants providing saliva samples in the HYENA study, by country and noise exposure at residence.

Country	Aircraft noise exposure [*L*_Aeq,24h_ (dB)]			No. of subjects
	< 50	≥ 50 to < 60	≥ 60	
United Kingdom	35	5	47	87
Germany	43	17	19	79
Greece	10	48	10	68
The Netherlands	23	16	23	62
Italy	19	27	12	58
Sweden	44	29	12	85
Total	174	142	123	439

**Table 2:** Linear regression coefficients for the relation between air traffic noise exposure and morning saliva cortisol levels among 439 subjects in six European countries.^a^

	All		Women		Men	
*L*_Aeq,24h_ (dB)	No.	Coefficient (95% CI)	No.	Coefficient (95% CI)	No.	Coefficient (95% CI)
Continuous per 5 dB^b^	439	0.25 (−0.17 to 0.66)	230	0.80 (0.26 to 1.34)	209	−0.33 (−0.88 to 0.22)
Categorical						
< 50^c^	174	—	97	—	77	—
≥ 50 to < 60	142	1.04 (−1.61 to 3.68)	77	2.16 (−1.26 to 5.59)	65	0.06 (−3.64 to 3.76)
≥ 60	123	1.83 (−0.90 to 4.35)	56	6.07 (2.32 to 9.81)	67	−2.00 (−5.61 to 1.61)

a All analyses adjusted for road traffic, country, age, sex (only for “All”), employment status, occupational status, medication use, BMI, alcohol, diet, remedy during night, and other noise sources in living environment.

b Rise in cortisol (nmol/L) per 5-dB increase in noise level.

c Reference category, arithmetic mean cortisol level: all = 19.13 nmol/L, women = 17.7 nmol/L, men = 20.92 nmol/L.

**Table 3:** Linear regression coefficients for the relation between air traffic noise exposure and morning saliva cortisol levels among 439 subjects in six European countries.^a^

	Women		Men	
Country	No.	Coefficient per 5 dB^b^ (95% CI)	No.	Coefficient per 5 dB^b^ (95% CI)
United Kingdom	40	2.23 (0.45 to 4.01)	47	0.52 (−1.00 to 1.99)
Germany	43	0.02 (−1.73 to 1.77)	36	0.11 (−1.74 to 1.97)
The Netherlands	29	2.34 (−1.25 to 5.93)	33	0.84 (−2.56 to 4.25)
Sweden	46	1.09 (−0.12 to 2.31)	39	0.05 (−1.25 to 1.34)
Italy	43	−0.08 (−2.95 to 2.79)	25	−0.85 (−4.06 to 2.36)
Greece	29	−0.36 (−2.35 to 1.64)	29	−1.10 (−3.30 to 1.10)

a All analyses adjusted for road traffic, country, age, employment status, occupational status, medication use, BMI, alcohol, diet, remedy during the night, and other noise sources in living environment.

b Rise in cortisol (nmol/L) per 5-dB increase in noise level.

**Table 4:** Linear regression coefficients for the relation between air traffic noise exposure and morning saliva cortisol levels with regard to employment status among 230 women in six European countries.^a^

	Aircraft noise (dB)					
	< 50		50–60		> 60	
Employment status^b^	No.	Coefficient^c^ (95% CI)	No.	Coefficient^c^ (95% CI)	No.	Coefficient^c^ (95% CI)
Retired	33	—	24	0.62 (−5.18 to 6.41)	24	5.38 (−0.57 to 11.33)
Other	17	1.62 (−4.94 to 8.18)	25	5.36 (−0.91 to 11.63)	15	3.90 (−3.27 to 11.07)
Employed	47	3.93 (−1.64 to 9.49)	28	7.87 (1.63 to 14.11)	17	16.23 (9.29 to 23.2)

a All analyses adjusted for road traffic, country, age, sex, occupational status, medication use, BMI, alcohol, diet, remedy during night, and other noise sources in living environment.

b Employment status is classified categorically. “Retired” includes retired participants. “Other” includes participants on sick leave, unemployed subjects, housewives, and students. “Employed” included both full-time and part-time employment as well as self-employed working from home.

c Linear regression coefficients for morning saliva cortisol level in nmol/L. Arithmetic mean cortisol level in the reference category = 19.67 nmol/L.
